# The Preconception Health and Health Behaviors of Australian First-Time Fathers: A Cross-Sectional Study

**DOI:** 10.1177/10901981251414635

**Published:** 2026-01-24

**Authors:** Tristan Carter, Danielle Schoenaker, Jon Adams, Amie Steel

**Affiliations:** 1 University of Technology Sydney, Sydney, NSW, Australia; 2 University of Southampton, Southampton, UK; 3 University of Southampton and University Hospital Southampton NHS Foundation Trust, Southampton, UK

**Keywords:** preconception, first-time fathers, health behaviors

## Abstract

Preconception health can be optimized through preconception care, which is considered an effective catalyst for behavior change prior to parenthood and is of paramount importance due to the influence that health behaviors can have on pregnancy and offspring outcomes. The preconception health and preconception health behaviors of males before they become fathers for the first time remain underexplored and are an emerging area of interest for public health and reproductive health research. This article is the first report and quantitative cross-sectional analysis of the national dataset pertaining to male health, Ten to Men, reporting data relevant to male health across the life course, during preconception. This report offers research foresight into the health behaviors (e.g., smoking or alcohol), health conditions, health consultations, medications, health information, and health literacy of Australian males prior to becoming a father (*n* = 572). The findings of this research support an undervalued albeit indispensable research area by providing up-to-date evidence-based information regarding paternal preconception health and health behaviors. This public health research with a focus on paternal preconception health behaviors and health behavior change can only strengthen the call for preventive health and offer preconception health and preventive knowledge about males for the research community and practitioners.

During the months to years prior to conception, known as the *preconception period*, care and support can be initiated or provided as part of biomedical, behavioral, and social health interventions for women and couples to optimize pregnancy and offspring outcomes (World Health Organization [WHO], 2012). Preconception care is considered an effective catalyst for behavior change prior to parenthood, and a strategy to manage any potential barriers to health to ensure optimal outcomes (Suto et al., 2025). Research suggests modifying and sustaining behavior change up to 12 months but at least 3 months prior to conception to optimize preconception health status (Dorney & Black, 2018; Lassi et al., 2014). However, the importance of preconception health extends beyond the 12 months prior to conception and is therefore also relevant to males during early childhood and adolescence who, as potential future fathers, can be engaged by other males to promote positive health behaviors which translate into adulthood (Gaynor et al., 2024).

Preconception health can be optimized through effective preconception care strategies to systematically address exposures and behaviors known to have epigenetic influences on health, such as smoking, excessive alcohol consumption, a nutritionally balanced diet (Day et al., 2016), and regular physical activity (Marcho et al., 2020). The paternal epigenetic influences on placental health and their impacts on offspring development and disease cannot be underestimated (Bhadsavle & Golding, 2022). Addressing these behaviors is a key consideration due to their known influence on adverse pregnancy and offspring outcomes, such as risk of preterm birth or low birthweight (Carter et al., 2023). Health conditions such as depression or asthma are also key considerations to be addressed during the preconception period due to their known influences on pregnancy and offspring outcomes such as infertility (Liao et al., 2024) or spontaneous abortion (Yland et al., 2022).

In addition to health behaviors and health conditions, social determinants of health (Australian Institute of Health and Welfare, 2022), alongside characteristics such as gender (Maas et al., 2022) and age (Nilsen et al., 2013), may influence an individual’s engagement and responsiveness to preconception care strategies and their ability to optimize health behaviors. Social determinants include characteristics such as being married or being in a committed relationship, being employed and able to sustain financial comfortability, or having stable housing (Steel et al., 2025), all of which will influence an individual’s health behavior’s and access to health services However, preconception care that supports males to optimize medical, behavioral, and social risk factors is currently an underutilized approach (Caut et al., 2022; Shawe et al., 2015; Toivonen et al., 2017).

A male’s roles, responsibilities, and contributions before conception are contingent on their awareness and motivation toward planning and preparing for pregnancy and parenthood (Shawe et al., 2019). This may be influenced by the engagement in pregnancy preparation of their female partner (Caut et al., 2022; Harlow et al., 2020), and the knowledge and expertise of health practitioners and health services accessed (or not) by males (Hogg et al., 2019; Ojukwu et al., 2016). By acknowledging that a male’s responsibilities and influence commence in the preconception period (Kotelchuck & Lu, 2017), there is a need to actively include male partners in parenthood preparation (Entsieh & Hallström, 2016), integrate males into preconception care, and advocate for coordinated paternal preconception care delivery. The integration of males into preconception-based discussions is rarely considered within clinical encounters regarding pregnancy or family planning (Hogg et al., 2019; Kizirian et al., 2018) or within preconception care policies, guidelines, and recommendations (Dorney et al., 2022; Shawe et al., 2015).

In light of the current gaps in research, the study reported here draws upon the largest national all-male-cohort study, Ten to Men (TTM) (Australian Government, 2022), to ascertain the preconception health status and preconception health behaviors of Australian males before they become first-time fathers. In addition, the study explored the associations between preconception health characteristics and becoming a first-time father among Australian adult males.

## Materials and Methods

### Study Design

**Figure 1.**
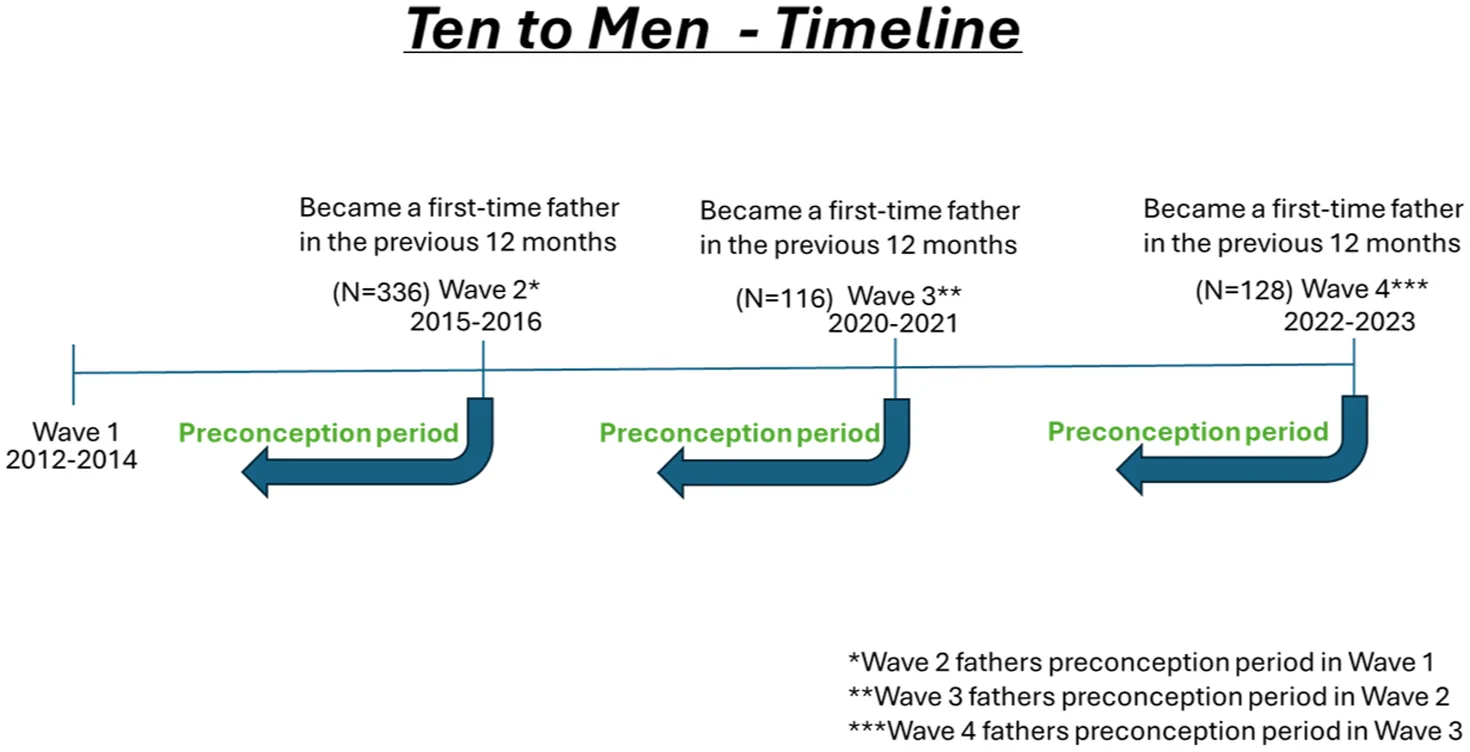
Ten to Men Timeline.

This study utilized longitudinal data from the Australian Longitudinal Study on Male Health (TTM) (Australian Government, 2022). TTM was conducted in four waves: Wave 1 [2012–2014], Wave 2 [2015–2016], Wave 3 [2020–2021], and Wave 4 [2022–2023] (see Figure 1). Access and use of TTM data (Version 4.0.1) were approved by the TTM Data Manager in accordance with the terms outlined in the TTM: The Australian Longitudinal Study on Male Health Data User Agreement and Deed of Confidentiality.

Study ethics approval was obtained from the University of Technology Sydney [UTS] Human Research Ethics Committee [HREC] (ETH24-10077). This study complies with the Reporting of Observational studies in Epidemiology [STROBE] guidelines (Cuschieri, 2019).

### Setting and Participants

TTM was conducted more than 8 years in four waves commencing in 2013 (Australian Government, 2022) and recruited approximately 24,000 boys and men aged from 10 to 55 years old, at Wave 1, from across Australia (Australian Government, 2022). Study recruitment used a stratified, multistage cluster random sample design which identified Australian males based upon clusters of geographical units (Australian Government, 2022). Study participants were chosen at random to ensure diversity in representation across all Australian States and Territories. All participants who completed Wave 1 were invited to participate in the subsequent waves. A separate questionnaire was administered for each wave. The males considered for the current study were adult participants aged at least 18 years old who became a first-time father in the 12 months prior to any wave.

### Survey Instrument

The questionnaires administered for each wave generally reported the same variables although nuances were reported at each timepoint. In each questionnaire, survey respondents answered, “in the past 12 months, have you become a father for the first-time?.” Respondents who answered “yes” to this question were identified as “first-time fathers.”

The analysis undertaken for the study reported in this paper draws upon the following sociodemographic variables: age, region of birth, marital status, private health insurance status, employment status, main industry of employment, highest qualification completed following high school, identification as an Aboriginal or Torres Strait Islander, home ownership status, and combined household income.

Variables also included were health and health behaviors such as smoking [current smoker and cigarettes per day], alcohol consumption [standard drinks per day and frequency in the last 12 months], sleep [hours/minutes per night during week/on weekend], body mass index [BMI], number of new female sexual partners in the previous 12 months, fruit and vegetable servings per day, usual sitting time on a workday/weekend [minutes], and physical activity in the previous week [number of times and times spent in minutes].

Variables relevant to health conditions and health literacy were also analyzed including serious personal injury, illness or surgery in the last 12 months, incapacity due to injury, specific heath conditions in the past 12 months [Eczema, Asthma, COPD, Chronic Bronchitis, Arthritis, Cataracts, Diabetes mellitus, Depression, Post-traumatic stress, other anxiety disorders, schizophrenia, high cholesterol, high blood pressure, heart attack, heart failure, angina, stroke, cancer, sleep apnea], and variables relating to health literacy [understanding and discussing information from a health care provider].

Other variables analyzed related to health consultations [professionals in the last 12 months and frequency of checkups with a GP], reasons why health care cannot be accessed, and drug use in the past 4 weeks and 12 months [Marijuana].

### Data Analysis

TTM data version 4.0.1 incorporated four separate data files (one for each wave). The data files for each wave were imported into the STATA18 statistical software (StataCorp LLC) and merged using the unique identifier for each study participant.

From the merged data set, variables of interest were identified across all waves, and binary variables were generated from categorical variables to determine participants at each wave who identified as a first-time father in the past 12 months (yes, no). First-time fathers identified in each wave had their health behaviors analyzed from the previous waves, that is, any first-time fathers identified in Waves 2, 3, and 4 had their health behaviors analyzed from Waves 1, 2, and 3, respectively (Figure 1).

Frequencies and proportions for all categorical variables of interest were reported according to being a first-time father. Statistical differences in variables were explored using chi-square tests with an α-value of .05. The mean and standard deviation were reported for all continuous variables, and *t*-tests were applied to test for statistically significant differences. Any missing variables or responses identified in the questionnaire, such as (a) Not asked, (b) Don’t know, (c) Refused to answer, and (d) Invalid response, were not included in the analysis.

Backward stepwise logistic regression was used to identify the most parsimonious model for characteristics of males predicting who became first-time fathers. All differences in variables measured by chi-square tests or t-tests, with a *p* value <.20, were included in the regression model. The variable with the highest *p* value was removed from the model then the model was tested and retested until the final *p* value for the model was ≤0.05. Any variables which were only answered by a subset of the population were not included in the logistic regression analysis, for example, the number of cigarettes smoked per day (smokers only).

## Results

Across the four waves, a total of 11,811 adult males responded to the question regarding becoming a first-time father in the past 12 months (referred to as all respondents). A total of 572 adult males had become a father for the first time (first-time fathers) and therefore had health behaviors in previous waves which were reported prior to them becoming a father, during preconception. A total of 11,239 did not become a first-time father during the study period (others).

### Sociodemographic Characteristics

For all respondents, about half were aged 45 to 55 years (32.0%) or >55 years (16.6%) (see Table 1). Most respondents were born in Oceania (83.1%), married (70.0%), employed (79.1%), and/or reported an income more than $99,000 per year (*n*=63.3%).

**Table 1.** Sociodemographic Characteristics of Australian Adult First-Time Fathers.

Characteristics	All (*n* = 11,811)	Became first-time father in the previous 12 months (*n* = 572)	Did not become a first-time father in the previous 12 months (*n* = 11,239)	*p* value
	*M*, *SD*	*M*, *SD*	*M*, *SD*	
**Age (years)**	42.0; (12.2)	35.9 (8.2)	42.3 (12.3)	<.001
	*n* **(%)**	*n* **(%)**	*n* **(%)**	
*18 to 24 years*	1,398 (11.8)	37 (6.4)	1,361 (12.1)	<.001
*25 to 34 years*	1,931 (16.3)	231 (40.3)	1,700 (15.1)	
*35 to 44 years*	2,723 (23.0)	226 (39.5)	2,497 (22.2)	
*45 to 55 years*	3,789 (32.0)	61 (10.6)	3,728 (33.1)	
*Aged greater than 55 years*	1,970 (16.6)	17 (2.9)	1,953 (17.3)	
**Region of birth**	**(** *n* **= 7,755)**			
*Oceania and Antarctica*	6,449 (83.1)	340 (82.9)	6,109 (83.1)	.01
*Europe*	545 (7.0)	19 (4.6)	526 (7.1)	
*Africa and Mid East*	197 (2.5)	9 (2.2)	188 (2.5)	
*Asia*	481 (6.2)	39 (9.5)	442 (6.0)	
*Americas*	83 (1.0)	3 (0.7)	80 (1.0)	
**Marital status**	**(** *n* **= 10,445)**			
*Married/de facto*	7,318 (70.0)	393 (73.8)	6,925 (69.8)	.001
*Separated but not divorced*	306 (2.9)	7 (1.3)	299 (3.0)	
*Divorced/Widowed*	415 (3.97)	7 (1.3)	408 (4.1)	
*Never married*	2,406 (23.0)	125 (23.5)	2,281 (23.0)	
**Private health insurance**	**(** *n* **= 11,655)**			
	7,275 (62.4)	388 (68.0)	6,887 (62.1)	.004
**Employment status**	**(** *n* **= 11,735)**			
*Employed*	9,291 (79.1)	473 (82.8)	8,181 (78.9)	.002
*Unemployed*	1,240 (10.5)	64 (11.2)	1,176 (10.5)	
*Neither working or looking for work*	1,204 (10.2)	34 (5.9)	1,170 (10.4)	
**Education—highest qualification completed following high school**	**(** *n* **= 10,957)**			
*Have not completed any qualification*	1,274 (11.6)	37 (6.5)	1,237 (11.9)	<.001
*Begun but did not complete any qualification*	1,023 (9.3)	55 (9.7)	968 (9.3)	
*Vocational Education -Trade/Certificate/Diploma*	5,165 (47.1)	235 (41.7)	4,930 (47.4)	
*University degree or higher*	3,095 (28.2)	214 (38.0)	2,881 (27.7)	
*Other*	400 (3.6)	22 (3.9)	378 (3.6)	
**Identify as Aboriginal or Torres Strait Islander**	**(** *n* **= 11,770)**			
	261 (2.2)	16 (2.8)	245 (2.1)	.334
**Home ownership status**	**(** *n* **= 11,405)**			
*Owned outright, or with a mortgage, or being purchased under a rent/buy scheme*	5,690 (48.8)	283 (50.3)	5,407 (49.8)	.01
Renting or pay board	4,769 (41.8)	252 (44.8)	4,517 (41.6)	
*Being occupied rent-free*	653 (5.7)	17 (3.0)	636 (5.8)	
*Other*	293 (2.5)	10 (1.7)	283 (2.6)	
**Combined household income**	**(** *n* **= 10,971)**			
*Low income (up to $37,500 per annum or up to $769 per week)*	755 (6.8)	31 (5.6)	724 (6.9)	.05
*Moderate income (between $37,501 and $99,000 or between $770 and $1,919 per week)*	3,273 (29.8)	144 (26.2)	3,129 (30.0)	
*High income (more than $99,000 or at least $1,920 per week)*	6,943 (63.3)	374 (68.1)	6,569 (63.0)	

When comparing the sociodemographic characteristics of first-time fathers during the preconception period with others, first-time fathers mean age was lower (mean 35.9; *SD* 8.2 vs. 42.3; *SD* 12.3, *p* < .001). Age reported by category revealed a high proportion of first-time fathers aged between 25 and 34 years old (40.3%) or 35 and 44 years old (39.5%) compared with others from the same age ranges; 25 to 34 years (15.1%) or 35 to 44 years (22.2%) (*p* < .001). A greater proportion of first-time fathers were born in Asia (9.5%) compared with others (6.0%), whereas a smaller proportion of first-time fathers were born in Europe (4.6%) than others (7.1%) (*p* = .01). Also, first-time fathers were more often married (73.8%) and/or employed (82.8%) than others (69.8% and 78.9%), respectively (*p* = .002). First-time fathers were also more likely to hold private health insurance (68.0%) compared with others (62.1%). A larger proportion of first-time fathers held a university degree or higher (38.0%) and/or generated a high income, being more than $99,000 per annum, (68.1%) compared with others; university degree or higher (27.7%) (*p* < .001); high income (63.0%) (*p* = .05). Furthermore, a slightly greater proportion of first-time fathers were paying rent or board (44.8%) compared with others (41.6%) (*p* = .01).

### Health and Health Behaviors

All respondents who were smokers (35.6%) reported smoking an average of nine cigarettes per day (mean 8.8; *SD* 9.4) (see Table 2). Close to half of all respondents reported alcohol consumption at least once a week (45.5%), but most respondents met the alcohol guidelines of no more than four standard drinks per day (80.6%). Most respondents also met the daily sleep requirements during the weekend (91.6%). The majority of all respondents met the recommended daily limits for sitting on a non-workday (94.1%). Similarly, a large proportion of all respondents met the weekly recommendations for physical activity, both moderate (60.8%) and vigorous (77.5%).

**Table 2.** Health and Health Behaviors of Australian Adult First-Time Fathers.

Characteristics	All (*n* = 11,811)	Became first-time father in the previous 12 months (*n* = 572)	Did not become a first-time father in the previous 12 months (*n* = 11,239)	*p* value
	*n* (%)	*n* (%)	*n* (%)	
	**(** *n* **= 7,026)**			
**Smoking (current smoker)**	2,505 (35.6)	102 (31.1)	2,403 (35.8)	.08
	**(** *n* **= 2,580)**			
	*M; SD*	*M; SD*	*M; SD*	*p* value
Smoking (cigarettes per day—currently)^a^	8.8 (9.4)	4.9 (6.6)	11.4 (8.8)	<.001
	*n* (%)	*n* (%)	*n* (%)	*p* value
	**(** *n* **= 10,806)**			
**Alcohol** Meet the guidelines of no more than 4 drinks on any day?	8,713 (80.6)	463 (85.7)	8,250 (80.3)	.002
**Alcohol (frequency in the last 12 months)**	(*n* **=** 11,620)			
*Every day*	286 (2.4)	8 (1.4)	278 (2.5)	.01
*At least once a week*	5,289 (45.5)	234 (41.2)	5,055 (45.7)	
*Once a month or less frequently*	6,045 (52.0)	326 (57.4)	5,719 (51.7)	
**Sleep** Meet recommendations getting at least 7 hours and up to 9 hours a day?				
	**(** *n* **= 11,147)**			
*Weekday*	8,305 (74.5)	393 (72.9)	7,912 (74.6)	.38
	**(** *n* **= 11,652)**			
*Weekend*	10,683 (91.6)	513 (90.6)	10,170 (91.7)	.35
	*M; SD*	*M; SD*	*M; SD*	*p* value
	**(** *n* **= 11,811)**			
**Body mass index (BMI)**				
Underweight (BMI <18.5)	194 (1.6)	2 (0.3)	192 (1.7)	.002
Normal weight (BMI 18.5 – 24.9)	1,613 (13.66)	91 (15.9)	1,522 (13.5)	
Overweight (BMI 25.0 – 29.9)	3,287 (27.8)	182 (31.8)	3,105 (27.6)	
Obesity (BMI>30)	6,717 (56.8)	297 (51.9)	6,420 (57.1)	
	**(** *n* **= 2,222)**			
	*n* (%)	*n* (%)	*n* (%)	*p* value
**Fruit serves per day** Meet the recommended daily intake of 2 or more serves?	9,365 (79.2)	465 (81.3)	8,900 (79.1)	.22
**Vegetable serves per day** Meet the recommended daily intake of 5 or more serves?	5,767 (48.8)	259 (45.2)	5,508 (49.0)	.08
Sitting time usual workday Meet the recommended daily limit of no more than 7 hrs?	10,452 (88.4)	494 (86.3)	9,958 (88.6)	.10
Sitting time usual non-workday Meet the recommended daily limit of no more than 7 hrs?	11,122 (94.1)	551 (96.3)	10,571 (94.0)	.02
**Physical activity [previous week]** *Moderate physical activity (time spent)*				
Meet the recommendations of at least 2.5 hours per week?	7,190 (60.8)	320 (55.9)	6,870 (61.1)	.01
**Physical activity [previous week]** *Vigorous physical activity (time spent)*				
Meet the recommendations of at least 1.25 hours per week?	9,160 (77.5)	469 (81.9)	8,691 (77.3)	.009
	**(** *n* **= 10,886)**			
	*M; SD*	*M; SD*	*M; SD*	*p* value
*Total time of physical activity in minutes (last 7 days)*	334.2 (301.3)	383.4 (299.3)	332.5 (301.1)	<.001
**Drug Use**				
**Marijuana in the past 4 weeks** ^b^	(*n* **=** 1,342)			
	674 (50.2)	32 (51.6)	642 (50.1)	.82
**Marijuana in the past 12 months**	(*n* **=** 11,336)			
	2,986 (26.3)	145 (25.5)	2,841 (26.3)	.65

a Question asked to current smokers only. ^b^ Only asked if used in the past 12 months.

First-time fathers more often reported being overweight before conception (31.8%) compared with others (27.6%) (*p* = .002) but generally smoked less cigarettes per day (mean 4.9; *SD* 6.6) than others (mean 11.4; *SD* 8.8) (*p* < .001). The proportion of first-time fathers who reported daily alcohol consumption (1.4%) was less than others (2.5%) (*p* = .01), and a greater proportion of first-time fathers met the alcohol guideline (*n* = 85.7%) compared with others (80.3%) (*p* = 0.002). The requirements for vigorous physical activity per week were also met by a greater proportion of first-time fathers (81.9%) compared to others (77.3%) (*p* = 0.009). First-time fathers also reported a greater average number of minutes of physical activity per week (mean 383.4; *SD* 299.3) than others (mean 332.5; *SD* 301.1) (p<0.001).

Fewer first-time fathers reported meeting the recommendations for moderate physical activity per week (55.9%) compared with others (61.1%) (*p*<.001).

### Health Conditions

More than one-quarter of all respondents (28.4%) had reported serious personal injury, illness or surgery in the previous 12 months and more than half of all respondents reported being partly limited by this injury (55.3%) (see Table 3). The most reported health conditions were depression (19.5%), other mental health conditions (21.0%), high blood pressure (15.8%), high cholesterol (13.9%), and asthma (12.9%). Less than one in 10 respondents reported the remainder of all other health conditions, such as eczema (10.7%), high blood pressure (9.8%), and sleep apnea (7.8%).

**Table 3.** The Health Conditions of Australian Adult First-Time Fathers.

Characteristics	All (*n* = 11,811)	Became first-time father in the previous 12 months (*n* = 572)	Did not become a first-time father in the previous 12 months (*n* = 11,239)	*p* value
	*n* (%)	*n* (%)	*n* (%)	
	**(** *n* **= 11,632)**			
**Serious personal injury, illness, or surgery (last 12 months)**	3,312 (28.4)	156 (27.3)	3,156 (28.5)	.53
**Limited by Injury** ^a^	(*n* **=** 2,915)			
*Yes—Fully*	221 (7.6)	5 (4.0)	216 (7.7)	.22
*Yes- but only partly*	1,612 (55.3)	67 (54.0)	1,545 (55.3)	
**Health conditions in the past 12 months**	(*n* **=** 11,423)			
*Eczema*	833 (7.2)	61 (10.7)	772 (7.1)	.001
	**(** *n* **= 11,769)**			
*Asthma*	1,523 (12.9)	79 (13.8)	1,444 (12.9)	.52
	**(** *n* **= 11,048)**			
*COPD*	89 (0.8)	3 (0.5)	86 (0.8)	.45
	**(** *n* **= 11,046)**			
*Chronic bronchitis*	188 (1.7)	9 (1.6)	179 (1.7)	.84
	**(** *n* **= 11,046)**			
*Arthritis*	1,126 (10.1)	28 (4.9)	1,098 (10.5)	<.001
	**(** *n* **= 11,046)**			
*Cataracts*	80 (0.7)	3 (0.5)	77 (0.7)	.58
	**(** *n* **= 11,728)**			
*Diabetes mellitus*	595 (5.0)	23 (4.0)	572 (5.1)	.24
	(*n* **=** 11,774)			
*Depression*	2,295 (19.5)	102 (17.8)	2,193 (19.6)	.30
	**(** *n* **= 11,047)**			
*Posttraumatic stress*	305 (2.7)	21 (3.7)	284 (2.7)	.15
	**(** *n* **= 11,724)**			
Other anxiety disorders or mental health conditions^b^	2,466 (21.0)	104 (18.2)	2,362 (21.1)	.09
	**(** *n* **= 11,426)**			
Schizophrenia	72 (0.6)	5 (0.8)	67 (0.6)	.44
	**(** *n* **= 11,730)**			
High cholesterol	1,639 (13.9)	44 (7.7)	1,595 (14.3)	<.001
	**(** *n* **= 11,726)**			
High blood pressure	1,860 (15.8)	56 (9.8)	1,804 (16.1)	<.001
	**(** *n* **= 11,044)**			
Heart attack	124 (1.1)	3 (0.5)	121 (1.1)	.17
	**(** *n* **= 11,046)**			
Heart failure	56 (0.5)	2 (0.3)	54 (0.5)	.60
	(*n* **=** 11,045)			
Angina	116 (1.0)	3 (0.5)	113 (1.0)	.21
	**(** *n* **= 11,727)**			
Stroke	76 (0.6)	2 (0.3)	74 (0.6)	.36
	(*n* **=** 11,729)			
Cancer^c^	349 (2.9)	5 (0.8)	344 (3.0)	.002
	**(** *n* **= 11,725)**			
Sleep apnea	1,029 (8.7)	45 (7.8)	984 (8.8)	.43

a Question only asked to respondents with an injury. ^b^ Other anxiety conditions separate from posttraumatic stress disorder. ^c^ Includes bowel cancer, bladder cancer, brain cancer, prostate cancer, skin cancer, testicular cancer, thyroid cancer, other, or unknown types of cancer.

To compare the health conditions of first-time fathers and others, several conditions were reported less often by first-time fathers including arthritis (4.9%) versus (10.5%) (*p* < .001); high cholesterol (7.7%) versus (14.3%) (*p* < .001); high blood pressure (9.8%) versus (16.1%) (*p* > .001); and cancer (0.8%) versus (3.0%) (*p* < .001). First-time fathers more often reported eczema (10.7%) versus (7.1%) (*p* = .001).

### Health Consultations, Health Information, and Health Literacy

The majority of all respondents had consulted a GP in the past 12 months (91.3%) and approximately one third of all respondents have consulted a specialist doctor (37.8%) or pharmacist (32.2%) during the same period (see Table 4). From the respondents who consulted with a GP, 44% reported the frequency of GP checkups was “never.” When exploring the reasons why health care in general could not be accessed, respondents could not access health care due to waiting times being too long or there being no appointments (34.8%), due to cost (34.2%), or simply due to not deciding to seek care (29.7%).

**Table 4.** The Health Consultations, Health Information, and Health Literacy of Australian First-Time Fathers.

Characteristics	All (*n* = 11,811)	Became first-time father in the previous 12 months (*n* = 572)	Did not become a first-time father in the previous 12 months (*n* = 11,239)	*p* value
	*n* (%)	*n* (%)	*n* (%)	
**Consulted with a health care professional in the last 12 months**				
	(*n* = 11,728)			
*General practitioner (GP)*	10,713 (91.3)	535 (93.7)	10,178 (91.2)	.04
	(*n* = 11,728)			
*Specialist doctor*	4,441 (37.8)	197 (34.5)	4,244 (38.0)	.08
	(*n* = 11,728)			
*Pharmacist*	3,781 (32.2)	204 (35.7)	3,577 (32.0)	.06
	(*n* = 11,728)			
*Chiropractor*	2,436 (20.7)	119 (20.8)	2,317 (20.7)	.96
	(*n* = 11,759)			
*Dentist*	7,264 (61.7)	364 (63.7)	6,900 (61.6)	.32
	(*n* = 11,080)			
*Diabetes educator*	192 (1.7)	10 (1.7)	182 (1.7)	.94
	(*n* = 11,728)			
*Naturopath*	427 (3.6)	19 (3.3)	408 (3.6)	.68
	(*n* = 11,080)			
*Social worker*	172 (1.5)	2 (0.3)	170 (1.6)	.01
**Frequency of consultations with a GP** ^a^ **(** *n* **= 11,711)**				
*More than once a year*	1,145 (9.7)	34 (5.9)	1,111 (9.9)	<.001
*Once a year*	2,177 (18.6)	73 (12.8)	2,104 (18.8)	
*Less frequently*	3,212 (27.4)	154 (27.0)	3.058 (27.4)	
*Never*	5,177 (44.2)	309 (54.2)	4,868 (43.7)	
**Reasons why healthcare cannot be accessed (** *n* **= 2,861)**				
*No service in area*	554 (19.3)	24 (15.5)	530 (19.6)	.22
*Waiting times too long/no appointments*	996 (34.8)	55 (25.7)	941 (34.7)	.80
*Not taking new patients*	230 (8.0)	18 (11.7)	212 (7.8)	.08
*Cost*	980 (34.2)	51 (33.1)	929 (34.3)	.76
*Decided not to seek care*	852 (29.7)	48 (31.1)	804 (29.7)	.69
*Too busy*	571 (19.9)	36 (23.3)	535 (19.7)	.27
*Transport*	152 (5.3)	4 (2.6)	148 (5.4)	.12
*Language problems*	25 (0.8)	3 (1.9)	22 (0.8)	.14
*Other reasons*	99 (3.4)	6 (3.9)	93 (3.4)	.76
Health Information and literacy	*n* (%)	*n* (%)	*n* (%)	*p* value
**Item 36—Can make healthcare providers understand my problems (** *n* **= 11,566)**				
Cannot do	168 (1.4)	7 (1.2)	161 (1.4)	.22
Difficult	1,276 (11.1)	50 (8.9)	1,226 (11.2)	
Easy	10,029 (87.4)	501 (89.7)	9,528 (87.3)	
**Item 38—Can discuss health concerns with a healthcare provider (** *n* **= 11,475)**				
Cannot do	166 (1.4)	6 (1.0)	160 (1.4)	.40
Difficult	1,251 (10.9)	53 (9.5)	1,198 (10.9)	
Easy	10,058 (87.6)	498 (89.4)	9,560 (87.5)	
**Item 41—Have a good discussion about your health with doctors (** *n* **= 11,478)**				
Cannot do	143 (1.2)	4 (0.7)	139 (1.2)	.34
Difficult	1,257 (10.9)	55 (9.8)	1,202 (11.0)	
Easy	10,078 (87.8)	499 (89.4)	9,579 (87.7)	
**Item 49—Can discuss things until you understand all that you need to (** *n* **= 11,471)**				
Cannot do	159 (1.3)	5 (0.9)	154 (1.4)	.50
Difficult	1,354 (11.8)	62 (11.1)	1,292 (11.8)	
Easy	9,958 (86.8)	491 (87.9)	9,467 (86.7)	
**Item 54—Can ask questions to get the health info that you need (** *n* **= 11,469)**				
Cannot do	152 (1.3)	5 (0.9)	147 (1.3)	.48
Difficult	1,220 (10.6)	54 (9.6)	1,166 (10.7)	
Easy	10,097 (88.0)	499 (89.4)	9,598 (87.9)	
**Item 48—Ease of getting health info you understand (** *n* **= 11,515)**				
Cannot do	113 (0.9)	4 (0.7)	109 (0.9)	.66
Difficult	1,071 (9.3)	48 (8.6)	1,023 (9.3)	
Easy	10,331 (89.7)	507 (90.7)	9,824 (89.6)	

a Only asked respondents who consulted with a GP.

Comparing the health consultations of first-time fathers with others, a slightly higher proportion of first-time fathers reported consulting with a GP (93.7%) than others did (91.2%) (*p* = .04). In contrast, a slightly lower proportion of first-time fathers reported consulting with a social worker (0.3%) compared with others (1.6%). More than half of the first-time fathers reported never consulting with a GP for a checkup (54.2%) compared with 43% of others who reported the same (*p* < .001). There were no statistically significant differences in health literacy.

### Associations of Preconception Health and Health Behaviors With Becoming a First-Time Father

The preconception health characteristics that were associated with becoming a first-time father in the previous 12 months are reported in Table 5.

**Table 5.** Multivariate Logistic Regression on Associations Between Preconception Characteristics and Becoming a First-Time Father (Adjusted Model).

Characteristics	Become a first-time father in the previous 12 months		
	aOR	95% CI	*p* value
*Alcohol* *(Standard drinks in a day)* *Meet the guidelines of no more than 4 drinks on any day?*	1.44	(1.09–1.91)	.009
*Moderate physical activity (last 7 days)* *Meet the recommendations of at least 2.5 hours per week?*	0.63	(0.52–0.76)	<.001
*Vigorous physical activity (last 7 days)* *Meet the recommendations of at least 1.25 hours per week?*	0.66	(0.48–0.90)	.01
*Average physical activity total*	1.00	(1.00–1.00)	<.001
*Eczema*	1.61	(1.20–2.14)	.001
*Cancer*	0.21	(0.08–0.52)	.001

Males who became fathers for the first time were less likely to meet the recommended guidelines for moderate physical activity (aOR = 0.63; confidence interval [CI] = [0.52, 0.76]) or the guidelines for vigorous physical activity (aOR = 0.66; CI = [0.48, 0.90]) than others who did not become first-time fathers. There was no difference in the average total physical activity time between males who became first-time fathers and others (aOR = 1.00; CI = [1.00, 1.00]). Men who became fathers for the first time were also less likely to report cancer (aOR = 0.21; CI = [0.08, 0.52]) compared with others.

Preconception health characteristics identified to be associated with becoming a first-time father included meeting the recommended guidelines for daily safe alcohol consumption (aOR = 1.44; CI = [1.09, 1.91]) alongside reporting eczema (aOR = 1.61; CI = [1.20, 2.14]).

## Discussion

This study draws on data from a longitudinal cohort to examine the preconception health, health literacy, and health behaviors of Australian males prior to becoming a first-time father. Health conditions and behaviors that may impact pregnancy and child health outcomes were reported in this study, being overweight or having eczema were preconception health characteristics which were more likely to be reported by males who became a first-time father than others. Preconception health research exploring the pregnancy and offspring outcomes of individuals with eczema is scarce yet regular prolonged paternal use of common eczema treatments (Zakhem et al., 2019), such as topical steroids to downregulate inflammation, may have adverse outcomes for the child (The Australasian College of Dermatologists, 2021; Zakhem et al., 2019) which are yet to be understood. In addition, a review of literature details some of the pregnancy and offspring outcomes when males are overweight (Carter et al., 2023) and acknowledges being overweight as a preconception risk factor for both the male (for conditions such as hypertension) and their offspring (for issues such as high birthweight) (Mutsaerts et al., 2014). Independent of sociodemographic and health-related factors, logistic regression analysis reveals males becoming a first-time father were more likely to meet the recommendations for safe alcohol consumption and less likely to meet the recommendations for moderate or vigorous physical activity or to report cancer before conception. These findings offer valuable insights into the health of males prior to parenthood.

One key finding from this study was the high proportion of males who consulted with a GP prior to becoming a father for the first time. GPs are widely recognized as having a key role in preconception care, both in terms of screening and management of health risks (Dorney & Black, 2018; Royal Australian College of General Practitioners [RACGP], 2021; Withanage et al., 2024). Consulting with a GP provides greater exposure to general and multidisciplinary preconception health and care opportunities before fatherhood. Cultural factors, such as Australian masculinity (Piatkowski et al., 2023), will influence the perceptions of the importance of paternal preconception health, so all health professionals (including GPs) can function as an interconnected network of providers (Mercer et al., 2014) and, in doing so, support Australian males during the preconception period. However, Australian GPs are gatekeepers to patient access to government funding for primary care consultations and subsequent health care via referral (Australian Government, 2024). As such, GPs can not only provide preconception care but can facilitate opportunities for Australian males to reduce the costs of accessing specialist and allied health professionals which can also deliver components of preconception care. Indeed, GPs are a key health system point of contact for most Australian men in the preconception period (Australian Bureau of Statistics [ABS], 2024) and indigenous health workers may provide cultural health context for males during preconception who identify as Aboriginal or Torres Strait Islander (Mercer et al., 2014). Yet, previous research reveals males do not always feel comfortable or have the inclination to discuss matters relating to preconception health and care with the GP (Cassinelli et al., 2023; Jackson, 2023; Maas et al., 2022). In the context of males as future fathers, preconception health care encounters with a GP may foster various sentiments. Swedish research, for instance, reports fathers can feel misunderstood by health professionals when seeking guidance for postnatal depression (Hammarlund et al., 2015). Conversely, GPs report not feeling confident or well-equipped to facilitate effective preconception care discussions with male patients (Hogg et al., 2019; Ojukwu et al., 2016) and therefore may not prioritize screening health risks, promoting health behavior change, or making referrals aimed at optimizing pregnancy and offspring outcomes by improving paternal preconception health. Therefore, males may be engaging with GPs and other health professionals but not necessarily regarding pregnancy preparation. With this in mind, the first steps toward effectively addressing the implementation of effective paternal preconception care require normalizing the inclusion and engagements of males when discussing preconception care and pregnancy planning.

Our analysis also reveals males who become a father for the first-time were more likely to meet the guidelines for daily safe alcohol consumption compared with others who did not become a first-time father. This finding is partly supported by the results of published literature reporting that while males may intend to avoid or limit alcohol during the preconception period, they still often consume alcohol regularly (Carter et al., 2025; Shawe et al., 2019). Romantic partner alcohol use can influence alcohol consumption of the other partner (Muyingo et al., 2020) so the safe alcohol consumption by males in this study could be a proactive approach to health in support of their partner who may not be consuming alcohol in preparation for pregnancy (Bello et al., 2025; Crawford-Williams et al., 2015). Many Australian males (73%) are married prior to fatherhood (Save the Children Australia, 2023) so partner support during the pregnancy planning period should be an important consideration of males (Foglabenchi et al., 2024). However, the role of reproductive partners to support the preconception health of males is underexplored in the literature (Davison et al., 2019).

Males must concurrently consider the direct influence and impact their excessive alcohol consumption or binge drinking—five or more alcohol beverages per sitting—can have on pregnancy and offspring outcomes (Carter et al., 2023; Luan et al., 2022; Zhang et al., 2020). Paternal preconception alcohol consumption is acknowledged as having adverse effects on offspring which may include outcomes such as the increased odds of microcephaly or a shorter mean anogenital distance (Carter et al., 2023). However, evidence reveals that many males are not fully aware of their roles and responsibilities before conception (Maas et al., 2022; McGowan et al., 2020; Shawe et al., 2019) and therefore remain unaware of the direct influence they can have on pregnancy and offspring outcomes which can include epigenetic influences from behaviors such as alcohol consumption (Bedi et al., 2020). Safe alcohol consumption (no more than four standard drinks per day) is an influential factor for males and their reproductive partners who can help males to translate their intentions to moderate alcohol into healthful behaviors. During the preconception period, safe alcohol consumption must be considered a key behavior to address in the context of intentions for pregnancy to mitigate risks of adverse pregnancy and offspring outcomes.

Our analysis also shows that males who became first-time fathers were less likely than other males to meet the recommendations for moderate physical activity and less likely to meet the recommendations for vigorous physical activity in the previous 7 days. The importance of physical activity in improving preconception health has been acknowledged (Prosen et al., 2021); however, the literature and guidelines that focus on preconception care and discuss physical activity often do not integrate males (Dorney et al., 2022; Toivonen et al., 2017). Therefore, research that provides a link between male physical activity during the preconception period and the influence on pregnancy and offspring outcomes is limited (Carter et al., 2023). Research relevant specifically to physical activity suggests that aerobic or vigorous physical activity can influence mental health (Schuch & Vancampfort, 2021; Smith & Merwin, 2021), body composition (Westerterp, 2018), and possibly even influence sleep quality and sleeping patterns (Alnawwar et al., 2023; Dolezal et al., 2017), all of which are relevant to preconception health and can have the potential to influence pregnancy and offspring outcomes (Carter et al., 2023; Freeman et al., 2025; Spry et al., 2020). Our study findings of expectant partners not meeting physical activity recommendations suggest that Australian males are not taking advantage of the opportunities during the preconception period regarding the benefits of physical activity for their health, with moderate and vigorous physical activity remaining inconsistent or absent. This finding also suggests males who intend to conduct physical activity to optimize their health (Carter et al., 2025) may not necessarily meet the recommendations for this kind of physical activity but may instead meet recommendations for other physical activities such as walking. Further research is needed to involve reproductive males as future fathers who can be targeted as participants to help better understand males during the preconception period and their beliefs and intentions toward moderate and vigorous physical activity.

Another noteworthy finding of this study is the overall proportion of Australian males prior to becoming first-time fathers who did not meet the daily recommendation for vegetable consumption. More than half of the Australian males in our study did not meet the recommendations for vegetable consumption is concerning as vegetable consumption is an important aspect of a balanced healthy diet, providing vitamins, nutrients, and fiber (Australian Government, 2025). Yet, the proportion of Australian males not meeting the recommendations for vegetable consumption in our study is considerably greater than other Australian males generally reported at only 3% (ABS, 2022). The importance of nutrition as a key health behavior relevant to pregnancy and offspring outcomes has been highlighted in a review of paternal preconception modifiable risk factors (Carter et al., 2023). Indeed, insufficient vitamins from vegetable consumption, such as folate, may underlie some issues relating to male reproductive health and fertility (Hoek et al., 2020). The finding that Australian males did not meet the daily vegetable consumption in this study is also pertinent to sperm quality and insightful to the finding that more than half of the first-time fathers in our study had obesity. Vegetable consumption can have effects on sperm quality, with a low total sperm count associated with low vegetable intake (Ricci et al., 2020). Inadequate vegetable consumption has also been reported more commonly among infertile men (Mendiola et al., 2009). Paternal preconception vegetable consumption, body weight, and infertility may be interrelated (Australian Government: Centre for Population, 2024; Hoek et al., 2022; Tully et al., 2024). Our findings that many Australian males do not report daily recommended vegetable consumption may signal an important opportunity for clinicians to pursue the food habits of males and to assess the behavioral control of males to maintain a healthy diet, and body weight, especially when planning a pregnancy, to ensure optimal pregnancy and offspring outcomes.

Some limitations constrained the study outcomes. The survey data utilized for this study were self-reported and were subject to recall bias (Coughlin, 1990). Also, due to the timing of the TTM study waves, the preconception period could not be defined within a specific timeframe but rather within the varying time periods between each study wave. Furthermore, iterations in survey responses over the four respective data waves did not allow for the research team to optimize the longitudinal nature of the data, which was aggregated and analyzed cross-sectionally. Nevertheless, this study utilizes data from the largest all-male longitudinal study in Australia to identify findings pertinent to the important but underresearched topic of male preconception health and care

## Conclusion

Most males in our study generally reported health behaviors before parenthood, which indicated some alignment to preconception care recommendations such as meeting the guidelines for safe daily alcohol consumption. However, participants also reported preconception risk factors that may impact pregnancy and child health outcomes, such as not meeting the weekly recommendations for physical activity or not meeting the daily recommendations for vegetable serves. The health conditions of Australian males may also present as a risk factor that may impact pregnancy and child health outcomes. One in five soon-to-be fathers reported a mental health condition, which also identifies the need for addressing aspects of mental health during preconception care. These findings highlight the importance of both research examining and subsequently public health initiatives addressing, where necessary, the preconception health behaviors of Australian males to mitigate health risks associated with pregnancy and offspring outcomes.
